# Highly efficient semiconductor modules making controllable parallel microchannels for non-compressible hemorrhages

**DOI:** 10.1016/j.bioactmat.2024.02.006

**Published:** 2024-02-23

**Authors:** Fengbo Yang, Xiaoli Jia, Chao Hua, Feifan Zhou, Jianing Hua, Yuting Ji, Peng Zhao, Quan Yuan, Malcolm Xing, Guozhong Lyu

**Affiliations:** a Engineering Research Center of the Ministry of Education for Wound Repair Technology, Jiangnan University, Affiliated Hospital of Jiangnan University, Wuxi, 214000, China; b Wuxi School of Medicine, Jiangnan University, Wuxi, 214000, China; c Medical School of Nantong University, Nantong, 226019, China; d Department of Critical Care Medicine, Affiliated Hospital of Jiangnan University, Wuxi 214000, China; e Burn & Trauma Treatment Center, Affiliated Hospital of Jiangnan University, Wuxi 214000, China; f State Key Laboratory of Oral Diseases & National Center for Stomatology & National Clinical, Research Center for Oral Diseases, West China Hospital of Stomatology, Sichuan University, Chengdu, 610041, Sichuan, China; g Department of Mechanical Engineering, University of Manitoba, Winnipeg, R3T 2N2, Canada

**Keywords:** Parallel microchannel, Thermoelectric semiconductors, Ice template, Hemorrhage

## Abstract

Nature makes the most beautiful solution to involuted problems. Among them, the parallel tubular structures are capable of transporting fluid quickly in plant trunks and leaf stems, which demonstrate an ingenious evolutionary design. This study develops a mini-thermoelectric semiconductor P–N module to create gradient and parallel channeled hydrogels. The modules decrease quickly the temperature of polymer solution from 20 °C to −20 °C within 5 min. In addition to the exceptional liquid absorption rate, the foams exhibited shape memory mechanics. Our mini device universally makes the inspired structure in such as chitosan, gelatin, alginate and polyvinyl alcohol. Non-compressible hemorrhages are the primary cause of death in emergency. The rapid liquid absorption leads to fast activation of coagulation, which provides an efficient strategy for hemostasis management. We demonstrated this by using our semiconductor modules on collagen-kaolin parallel channel foams with their high porosity (96.43%) and rapid expansion rate (2934%). They absorb liquid with 37.25 times of the own weight, show 46.5-fold liquid absorption speed and 24-fold of blood compared with random porous foams. These superior properties lead to strong hemostatic performance in vitro and in vivo.

## Highlights

• Highly efficient thermal control.
• Mini-semiconductors device.
• Aligned micro-channels for super-hemostasis.
• Universal fabrication for most polymers.
• Injectable and shape memory.

## Graphical abstract

Arranging mini-semiconductors for aligned micro-tubular structures for hemostasis applications.

**Figure:**
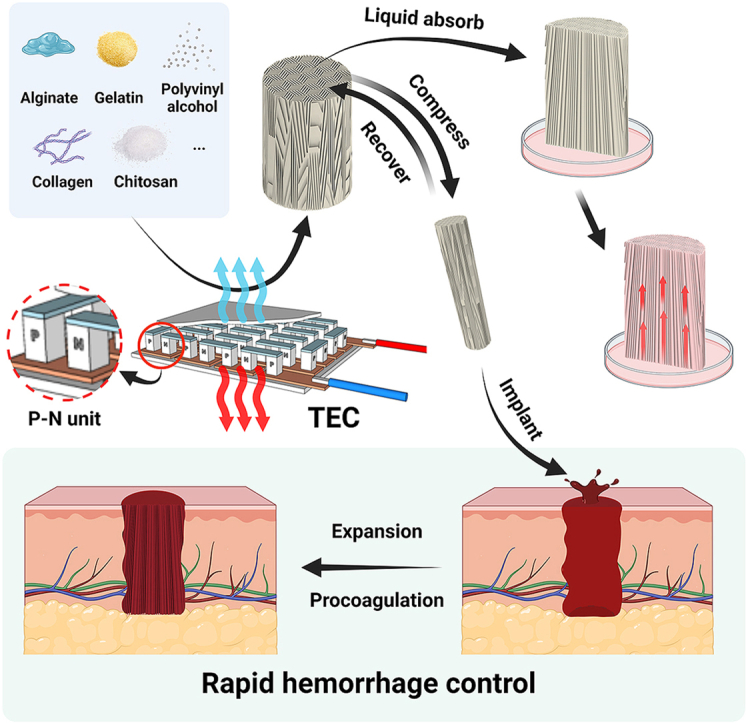
Scheme 1: Universal strategy for parallel channels of hydrogels by assembled mini-semiconductor modules and their application in hemorrhage control.

## 1 Introduction

Specific evolved parallel pipe structures, such as parallel microstructures, are geometrical structures with outstanding fluid management. In most plants, the vascular structure, formed by the xylem and bast fiber arranged in bundles, is essential for the transport of water and nutrients [[1], [2], [3]]. In animals, parallel renal tubules are typical structures for regulating transport. Efficient fluid concentration, reabsorption, and waste excretion are achieved through several parallel-aligned ducts exposed to gradient tissue osmotic pressure. Compared to isotropic foams with random pores, aligned materials with lower tortuosity have a stronger capillary effect as well as a lower liquid flow resistance, which increases the liquid absorbing speed [4]. This is particularly true for viscous liquids, such as tissue fluids or blood. The driving force for the continuous bottom-up capillary transport of water in the plant is mainly the negative pressure in the ducts caused by leaf transpiration and the osmotic pressure created by the active concentration of ions in the root system [5,6]. The absence of these endogenous dynamics can significantly reduce the performance of fluid transport. The gradient structure is also a significant physiological feature that plays a specific role. Apart from better mimicking the structure of the native tissues and concurrently meeting both biological and mechanical requirements [7], the Laplace pressure due to the gradient structure brings about additional dynamics in the capillary transport of liquids [8]. However, developing an eco-friendly and effective strategy to create aligned foams remains a challenge. Freeze casting is a method for controlling the directionality of a porous material by using the highly aligned solidification behavior of a solvent in a well-dispersed slurry [9]. Compared with other technologies (Table 1), ice template technology has several advantages, such as relatively low cost, moderate equipment requirements and process complexity, wide range of applications, and environmental sustainability. Nonetheless, the traditional freezing casting mode still has a significant level of equipment expenditure and relies on the cooling of liquid nitrogen or a compressor with freon refrigerant, which may face operational risk and climate change concerns [[10], [11], [12]]. Moreover, the interference and crosstalk between ice columns [9] induced by uneven freezing temperatures impede extensive applications.

**Table 1:** A summary of the main preparation methods and characteristics of oriented materials.

Technique		Materials	Characteristics	Limitations
Thermoelectric semiconductor module (Our work)		Universal materials, e.g., collagen; gelatin; chitosan; alginate; polyvinyl alcohol; kaolin	Environmental friendliness; miniaturization and modularization; efficient and cost-effective design; accurate temperature control	
Mechanical shear field	Doctor-blading /Tape casting	Ti_3_C_2_T_X_/hexamethylene [13]; Glycol mono-dodecyl ether [14]	Continuous production; simplicity and low cost	2D thin films/coatings
	Microfluidics	Regenerated cellulose [15]	Continuous fiber; core/shell configuration;	1D fibers; low-viscosity solutions
	Shearing microlithography	Graphene oxide [16]; graphite oxide [17]	High-precision control of vertically oriented structures; programmable designability	Complicated manufacturing process
Magnetic Field		Iron oxide nanoparticles [18]	Variable orientation direction; modulated spatial distribution	High magnetic field; long exposure time; additional magnetically responsive functional units
		Cobalt-doped titanium oxide [19]		
Electric Field		Silver-coated cellulose fiber [20,21]; Graphite/BaTiO3 [22]	Depending on strength of the electric field, the intrinsic conductive and dielectric characteristics	Higher electric field for insulating or surface modification
3D Printing		Pure Cr [23]; Direct-ink-writing [24,25]	Low volume; precision scale; customizable production; design freedom; environmentally sustainable; fabricate vertically aligned structures at a multi-scale level	Requirements for printing ink (shear thinning; rapid self-healing); long time
Hydrothermal Template		Titanium dioxide (TiO2) [[26], [27], [28], [29]]	Controllable dimension; exquisite configuration; high vertical orientation	Low yield; complex procedures; high energy consumption
Template-Directed Deposition		Long-range ordered vertical CNT arrays [30]		
Ice Template		Poly(vinyl alcohol) [31]; Boron nitride nanosheets [32]; Polyimide/Ti_3_C_2_T_x_ [33]	Flexible morphology control by freezing parameters; moderate equipment requirement and process complexity; wide application range	Customized equipment expenditure; environmental protection and safety hazards; refrigerant consumption; unstable quality control

The thermoelectric cooler, a solid-state active heat pump, works on the principle of the Peltier effect; when the direct current passes through the electric couple formed by P–N semiconductors in series, the two ends of the electric couple absorb and release heat [34]. Owing to the advantages of high reliability, noiseless operation, and accurate temperature control [35,36], thermoelectric cooling demonstrates huge potential in freeze casting with all the characteristics mentioned, because the arrangement and distribution of the porous characteristics are significantly influenced by the freezing speed and temperature. Consequently, by combining the thermoelectric device with the freezing casting theory, this study established a universal method for designing a gradient and parallel porous foam with high liquid absorption and excellent shape memory. In contrast to other manufacturing methods, our design shows wide applicability to materials (e.g., collagen, gelatin, chitosan, alginate and polyvinyl alcohol). From natural biological materials to synthetic polymer materials, there are many types of raw materials that can be flexibly customized in accordance with the application requirements of different microenvironments.

**Fig. 1:**
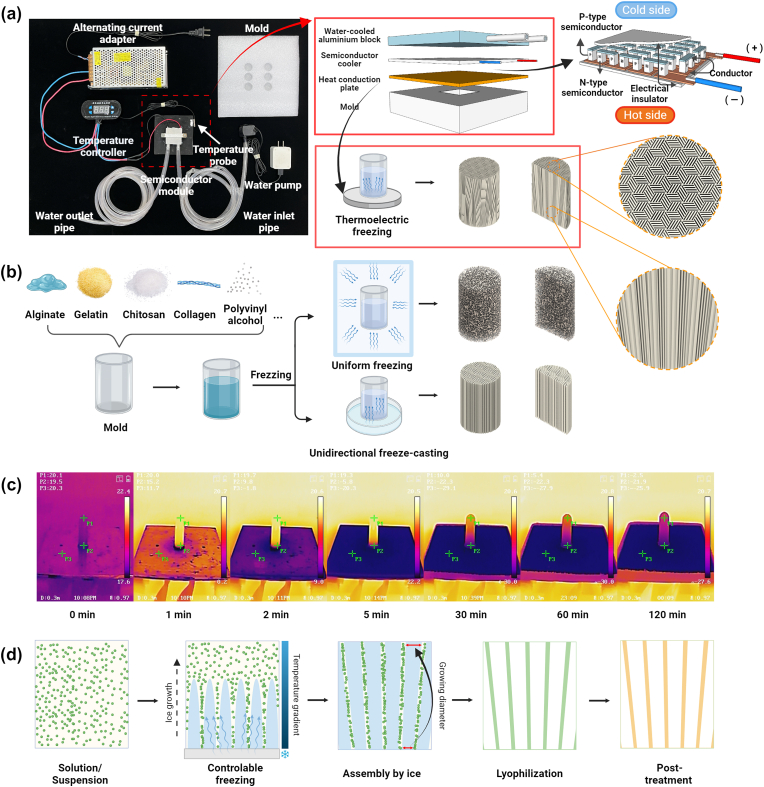
**Comparison of different freezing methods and the structural gradient characteristics in thermoelectric freezing.** (a) Thermoelectric semiconductor module for the gradient and parallel channeled foams. (b) The polymer slurry subjected to uniform freezing and unidirectional freeze-casting obtained porous foams with different structures. (c) Temperature distribution in materials during freezing via dynamic infrared thermography. (d) The process of ice crystal formation in thermoelectric freezing.

**Fig. 2:**
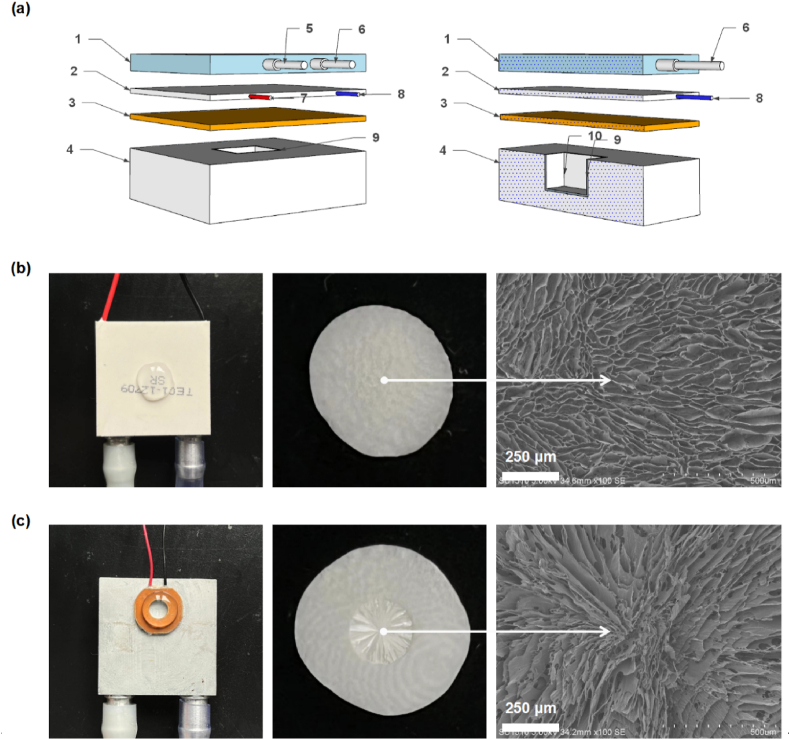
**Structure of freezing module and influence of semiconductor modules distribution on material pattern structure.** (a) Diagram of the freezing module. (1. Water-cooled block; 2. Semiconductor cooler; 3. Heat conduction plate; 4. Thermal barrier box; 5–6. Water inlet and outlet pipes; 7–8. Power cords; 9. Mold; 10. Loading well). (b) Rapid freezing of collagen droplets on common thermoelectric semiconductor module. (c) Rapid freezing of collagen droplets on circular thermoelectric semiconductor module.

**Scheme 1:**
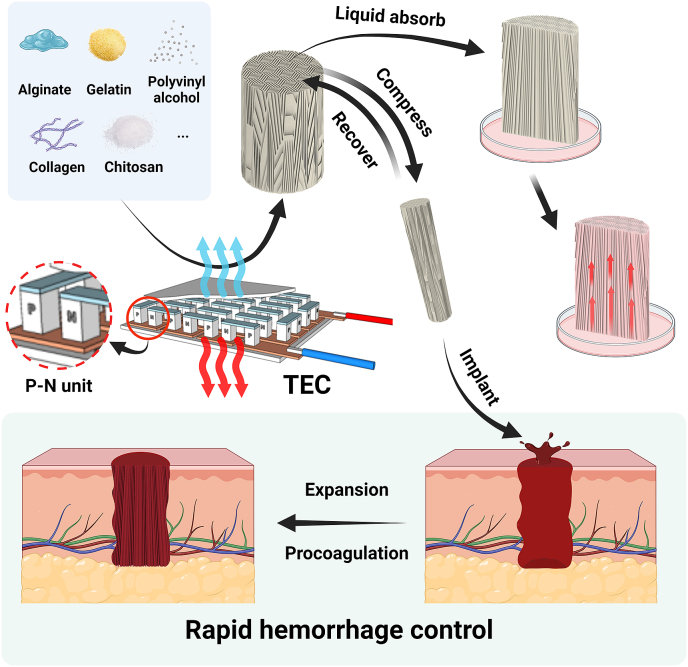
Universal strategy for parallel channels of hydrogels by assembled mini-semiconductor modules and their application in hemorrhage control.

Hemorrhage control is still a challenge and early and effective bleeding management after severe trauma is crucial to improve survival rate [37,38]. During coagulation, fast blood absorption indicates early coagulation activation with a large contact area between the material and blood, promoting the formation of blood clots [39]. In addition, the clearance of excess blood ensures direct and close contact between the material and the wound foundation [40]. Therefore, it is meaningful to prepare a hemostatic material with a parallel-tubular structure (Scheme 1). With regard to non-compressible wounds, current hemostasis device has long been concerns in low blood absorption efficiency and slow shape recovery of existing hemostatic materials for irregular wounds. In this study, we developed a new strategy for parallel microtubular structures which can have an enormous hemostasis potential for irregular shape and incompressible wound hemostasis.

## 2 Results and discussion

### 2.1 Preparation and structural characteristics of aligned foams

Conventional freeze-drying yields a random porous structure, whereas P–N conductors quickly form temperature gradience to bring in aligned channels (Fig. 1a and b). Though similar study has been done by using liquid nitrogen (LN) as refrigerants, poor controllability and fixed LN temperature limit its application scenarios [39,41]. Therein, a semiconductor module has obvious advantages over others. Direct electrothermal conversion ensures accuracy and controllability of temperature, besides economics and convenience. Within 5 min, our device can make a temperature transition from 20 °C to −20 °C in polymer solution (Fig. 1c). Unidirectional heat transfer ultimately contributes to the temperature gradient distribution of the system which ultimately affects the structure [42].

**Fig. 3:**
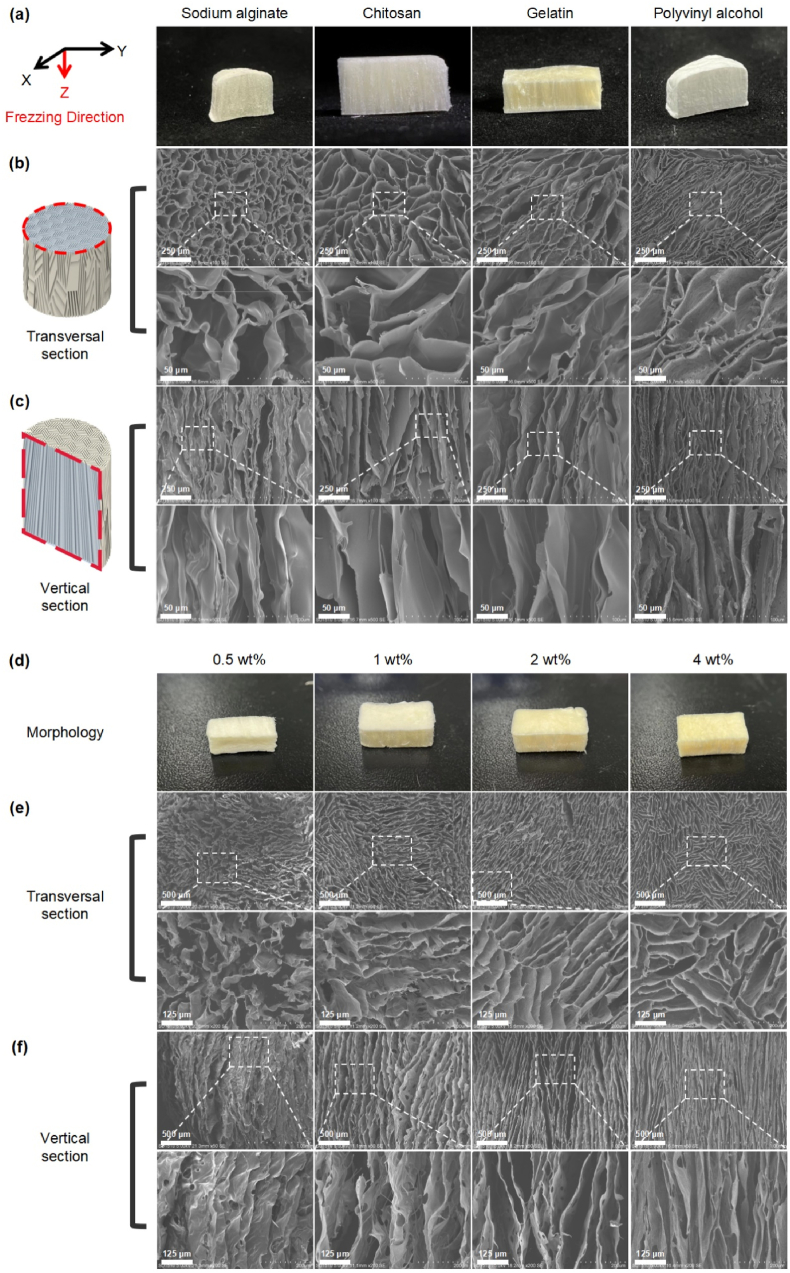
**Morphology and microstructure of foams fabricated by thermoelectric freezing with various materials or different material concentrations.** (a) The growth direction (Z-axis) of the ice crystals in the 3D coordinate system and morphology of the paralleled microtubular foams originated from sodium alginate, chitosan, gelatin and polyvinyl alcohol. (b–c) Scanning electron microscope (SEM) images of the paralleled microtubular foams in transversal and vertical sections. (d) Morphology of collagen foams with 0.5, 1, 2 and 4 wt% (from left to right). (e–f) SEM images of collagen foams with different concentrations along transversal and vertical sections.

**Fig. 4:**
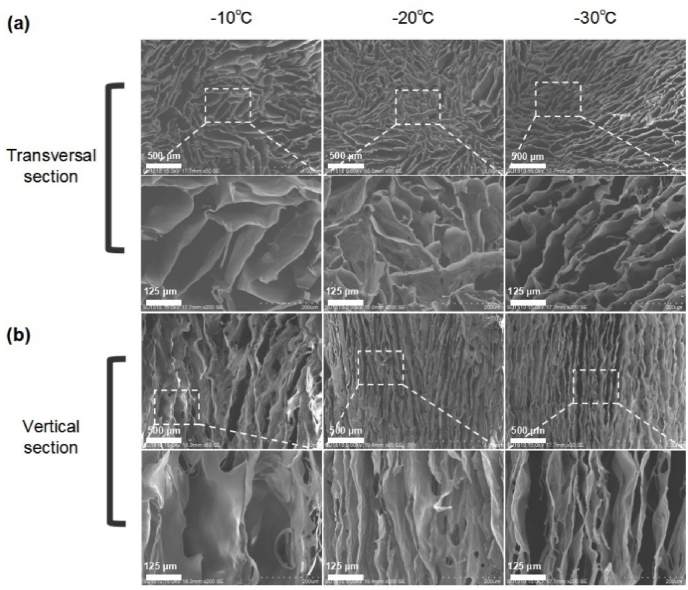
**Structures of foams made by thermoelectric freezing under different temperature gradients.** (a–b) SEM images along transversal and vertical directions.

**Fig. 5:**
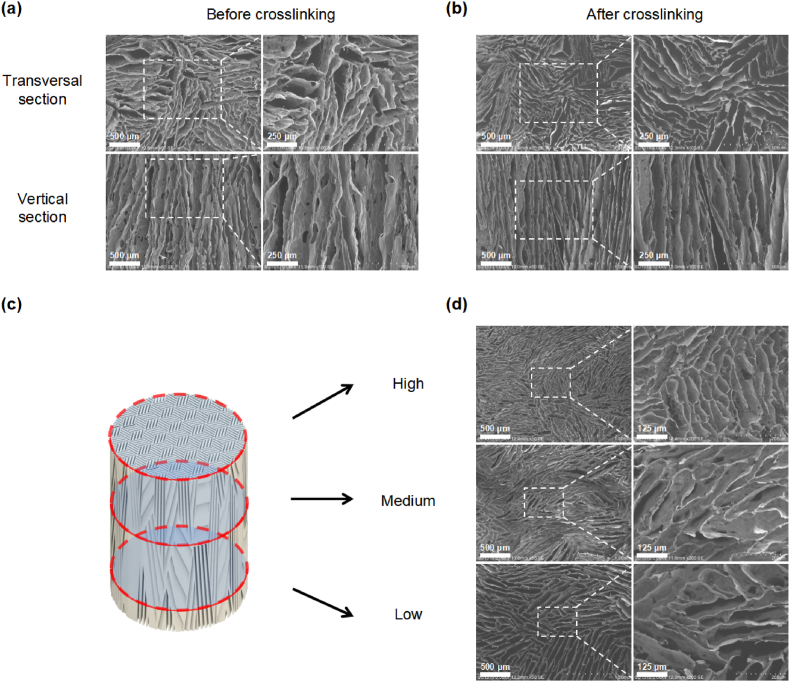
**Effect of cross-linking on structure and pore distribution in foams.** (a–b) SEM images of the paralleled microtubular foams of 2% collagen before (a) and after (b) vapor crosslinking with glutaraldehyde (GA). (c–d) Transversal SEM sections along the vertical directions.

Under this P–N conductor module, the unidirectional freezing of polymer solution instantly condense liquid into ice crystals. The crystals then propagate and expand along the temperature gradient. On the low temperature side, higher supercooling and solidification rate occur, which induces the formation of narrow and thin-walled pores. While the advancement of the water-ice front results in a reduction of the degree of supercooling preceding the solidifying interface, leading to an increase in the radius of the ice crystals [9,42]. When freezing, the solute is confined to the voids between the ice crystals, resulting in the development of a lamellar microstructure that is oriented parallel to the direction of the freezing front [42,43]. A small proportion of solutes are entrapped within the ice crystals, which facilitate the creation of inorganic bridges between adjacent walls. Ultimately, a gradient channeled foam of up to 40 mm is formed (Fig. S1). After lyophilization and ice crystals’ sublimation, the porous foam with gradient characteristics is generated (Fig. 1d). Interestingly, patterned structure can be formed radially and be determined by the distribution of small semiconductor modules (Fig. 2, Movie S1–S2), implying potential application where anisotropic but orderly structure is considered. Moreover, a series of radially oriented foams have been prepared based on biological or synthetic materials (Fig. 3a–c). All sponges exhibited small-scale lamellar patterns in the cross-section with general isotropic in cross-section area; while in the vertical directional, the structure presented parallel. Thus, overall, it is of anisotropic. This structure endows the foam with allotropic mechanism and oriented fluid flow. The concentration of the precursor and freezing temperature were two primary factors determining the wall thickness and channel width/pore size [4]. For collagen, a concentration-dependent relationship was observed for the channel width. When collagen concentration increased from 0.5% to 4%, the channel width decreased considerably ranging from 83.80, 72.96, 52.05, and then to 34.64 μm (Fig. 3d–f). Similarly, control of the freezing temperature was effective in regulating the mean channel width, ranging from 113.25 to 53.81 μm (Fig. 4). Meanwhile, no obvious changes were observed in the orientation and pore size after crosslinking (Fig. 5a and b). In the paralleled microtubular foam, gradient distribution of the freezing temperature allowed the pore size to range from top of 33.0 μm (low temperature end) to bottom of 82.4 μm (high temperature end) (Fig. 5c and d).

Supplementary video related to this article can be found at https://doi.org/10.1016/j.bioactmat.2024.02.006

The following is the supplementary data related to this article:

Movie S1 Movie S1

Movie S2 Movie S2

### 2.2 Design and characterization of collagen hemostatic foams

The aligned structure inspires us to consider a design for rapid liquid-triggered shape recovery and directional liquid transport: an injectable shape memory hemostatic dressing. Collagen and kaolin were used for the proposed device for their well-recognized hemostatic function. The mechanical properties of materials have a significant correlation with their biological application, particularly with regards to concentration. In the context of hemorrhage treatment, it is imperative to administer a moderate and consistent compression upon injection into the wound to prevent inadequate hemostasis, as well as tissue ischemia and necrosis resulting from excessive pressure.

**Fig. 6:**
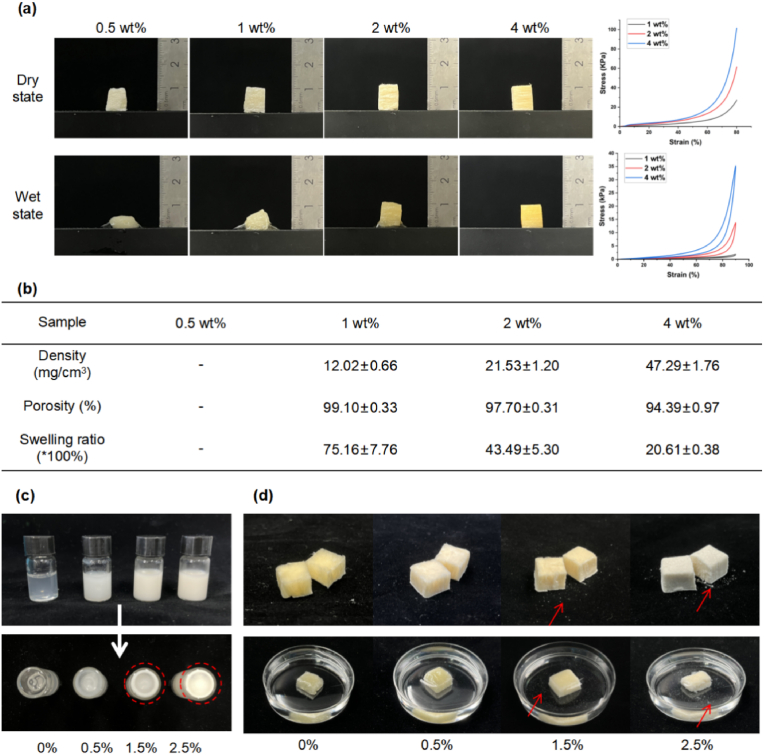
**Composition optimization of collagen hemostatic foam.** (a) Gross observation and compression stress-strain curves of foams with different collagen concentrations at dry and wet states in the transverse direction. (b) Density, porosity and swelling ratio of foams (n = 3). (c) The suspension stability of collagen solutions with different concentration of kaolin. (d) The stability of foams with different concentration of kaolin.

Here, foams with concentrations of 2 wt% or greater demonstrated favorable morphology in both dry and wet states, whereas 0.5 wt% and 1 wt% foams experienced challenges in maintaining effective mechanical support due to volume collapse (Fig. 6a). All foams exhibited porosity exceeding 95%, indicating high compression ratio and exceptional liquid absorption capacity (Fig. 6b). Overall, 4 wt% foams were then excluded due to its relatively low swelling ratio (20.61) and inadequate compression rate. Consequently, only foams of 2 wt% were subjected to further study. Considering the stability and safety of the material, the doping concentration of kaolin has to be optimized (0.5%, 1.5%, and 2.5% w/v). With an increase in kaolin concentration, particle sedimentation in the precursor solution became more pronounced (Fig. 6c), leading to uneven distribution in the foams and heightened risk of free particles entering the bloodstream (Fig. 6d). Ultimately, 0.5% kaolin was chosen due to its superior stability and dispersibility. According to the components, the final products were designated as 2% Col and 2% Col-k.

Fourier-transform infrared spectroscopy (FTIR) (Nicolet iS10, Thermo Fisher Scientific, USA) of 2% Col and 2% Col-K foams were studied and compared with those of kaolin powder and soluble collagen foam (Fig. S2). For 2% Col, three characteristic collagen absorption peaks, amide bands I, II, and III, were identified at 1629 (C=O) cm^−1^, 1547 (N–H) cm^−1^, and 1238 (C–N) cm^−1^. Moreover, owing to the stretching vibration of O–H and N–H bonds, the amide band A and B peaks of collagen were found at 3298 cm^−1^ and 3070 cm^−1^ [44]. Amide bands A and B, as well as amide bands I, II, and III, showed well-preserved collagen triple helix structures in 2% Col. For 2% Col-K, the addition of kaolin resulted in significant peaks at 1012 and 1008 (Si–O–Si) cm^−1^. New peaks were observed at 912 (Al–OH) cm^−1^ and 537 (Si–O–Si) cm^−1^ [[45], [46], [47]]. All the above results confirmed the successful loading of kaolin into the collagen foam.

### 2.3 Shape memory, mechanical performance and injectability of the aligned foams

**Fig. 7:**
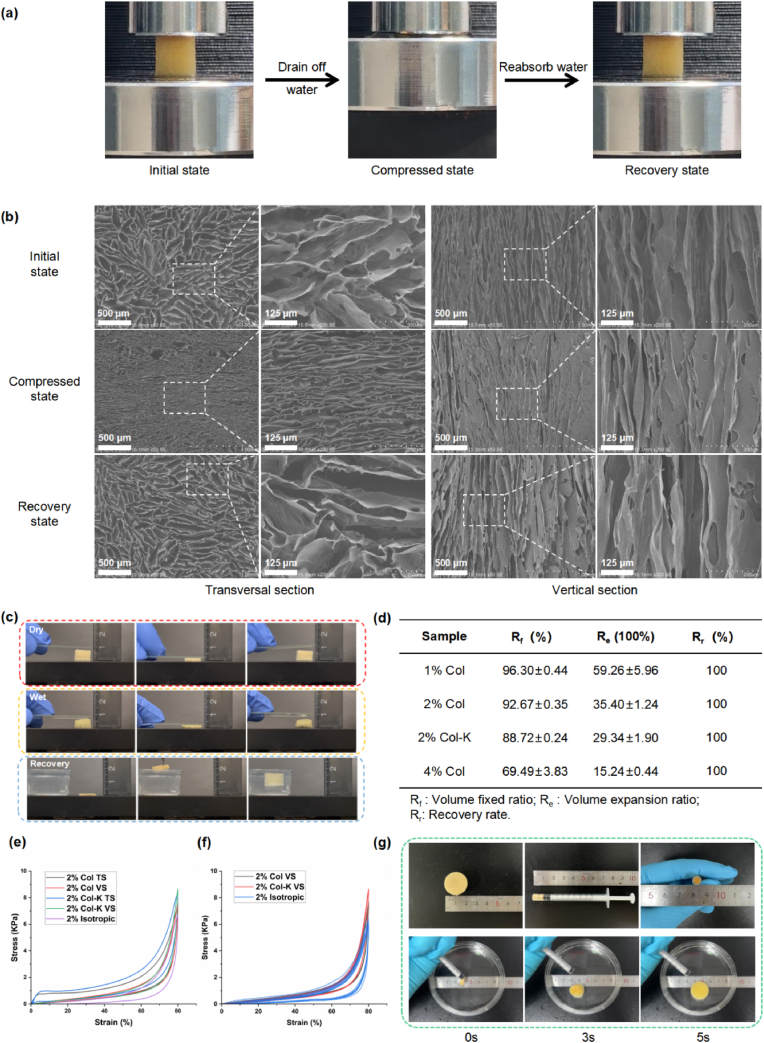
**Mechanical and shape memory properties of the foams.** (a) The shape memory cycle of the foam. (b) Micrography of foams in initial, compressed, and recovered shapes. (c) Compression-recovery performance of foams in dry and wet states. (d) Volume fixed ratio, volume expansion ratio, and recovery rate of shape memory capability of foam triggered by water. (n = 4) (e) Uniaxial compression stress−strain curves of 2% Col 2%, Col-K and 2% isotropic foam in transversal section and vertical section. (f) The stress–strain cycling curves of 2% Col, 2% Col-K and 2% isotropic foam with compression strains of 80% for 10 cycles in vertical section. (g) Evaluation of injection performance of foams.

In our research, the parallel foam exhibited recoverable compressibility in the transverse orientation (Fig. 7a, Movie S3). When compressed, the structure collapsed, with free water flowing out. When the stress was removed, spontaneous liquid reabsorption contributed to structure recovery. When the free water was completely absorbed, the shape under pressure remained unchanged. SEM observation was applied to the foam at the initial shapes, compressed shapes, and recovered shapes after fixation (Fig. 7b). As expected, the characteristic parallel structure of the foam was well-preserved during compression. No difference in the channel width before and after compression was observed. In the wet state, when water molecules penetrated the foam, the strength of the material decreased, while the toughness was greatly enhanced, avoiding damage to the structure under pressure (Fig. 7c). In this case, the reversible recovery of the porous foam was feasible. In summary, the foam demonstrated high liquid-triggered shape memory capability.

Supplementary video related to this article can be found at https://doi.org/10.1016/j.bioactmat.2024.02.006

The following is the supplementary data related to this article:

Movie S3 Movie S3

We further evaluated the shape-memory properties of the aligned foams qualitatively and quantitatively from the aspects of the volume fixed ratio, volume expansion ratio, and recovery rate. As shown in Fig. 7d, most foams exhibited high shape fixation (96.30, 92.67, and 88.72%) and volume expansion ratios (59.26, 35.40, and 29.34 *100%), except for 4% Col (69.49% volume fixed ratio and 15.24*100% volume expansion ratio). Once in contact with water, all the foams immediately returned to their original shape in less than 1 s (100% recovery). For the compressive performance, the parallel foams showed considerable directional differences in mechanical properties. In oriented foams, both 2% Col and 2% Col-K displayed higher compression modulus and isotropic foams had the smallest modulus at the same concentration (Fig. 7e). In in vertical section (VS), the soft stiffness but elasticity ensured the rapid compression performance. The addition of kaolin could also improve the compressive stress of parallel foams in both transversal and vertical direction. In addition, dynamic stress-strain tests (10 cycles at 80% strain) were performed to determine the compression fatigue resistance. Compared with isotropic foams, the oriented foams showed significantly better fatigue resistance (Fig. 7f).

The aligned foam with high stiffness in the axial direction facilitated delivery in a narrow space. In this regard, we loaded 20 mm diameter foam into a 5 mm inner diameter syringe and performed an injection demonstration. The shape of the material recovered quickly and completely within 5 s (Fig. 7g, Movie S4). Therefore, shape-memory agents have unique properties in hemostatic applications, where they are used in direct contact with an injector in a shape-fixed state deep into the base of the trauma and return to their original form upon contact with and absorption of blood. They are highly expected to completely fill the irregular wound in a short period, compressing the wound, and greatly reducing bleeding, especially in penetrating and gunshot wounds.

Supplementary video related to this article can be found at https://doi.org/10.1016/j.bioactmat.2024.02.006

The following is the supplementary data related to this article:

Movie S4 Movie S4

### 2.4 Liquid absorption capacity and hydrophilicity

**Fig. 8:**
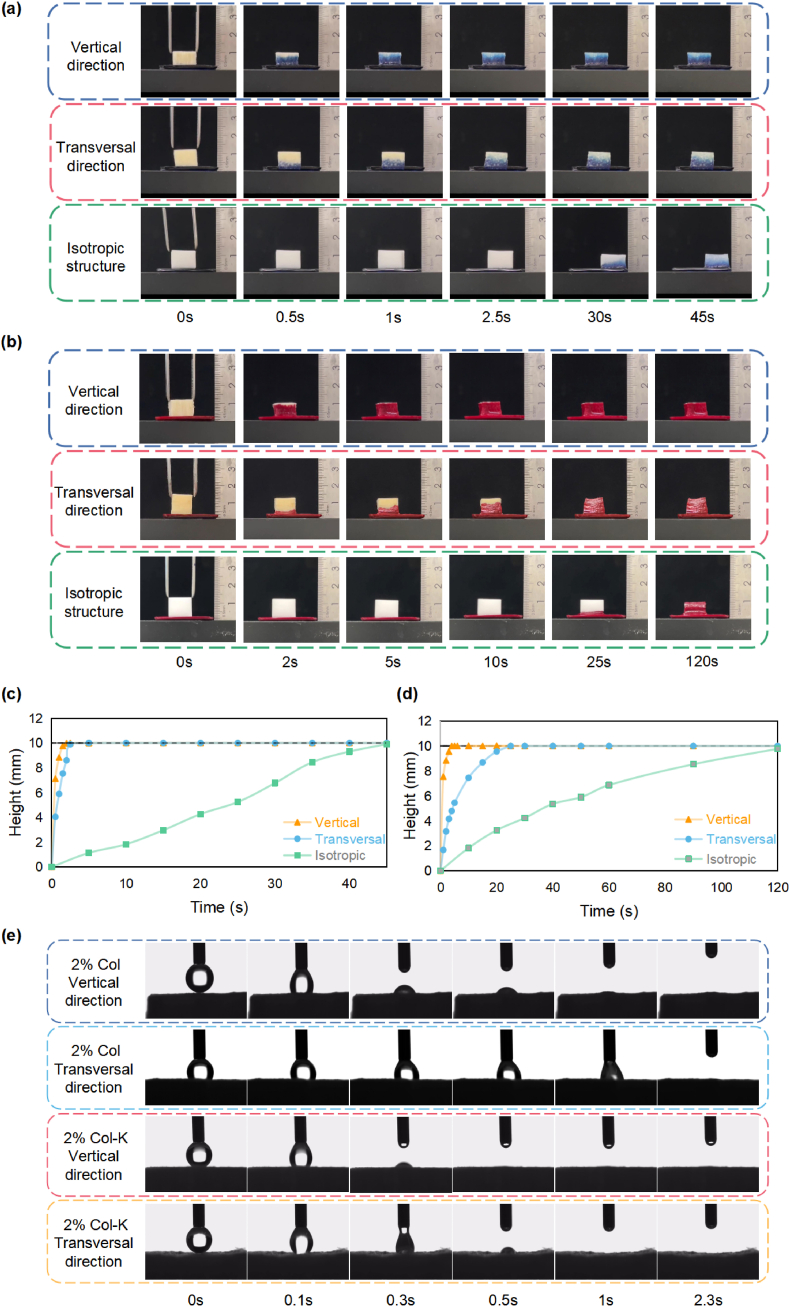
**Liquid absorption properties of the foams.** (a) Fluid absorption of the aligned foam in vertical direction and transverse direction using methylene blue solution. (b) Fluid absorption of the aligned foam in vertical direction and transverse direction using isotropic foam in blood. (c) The height of the absorbed methylene blue solution versus time. (d) The height of the absorbed blood versus time. (e) Water contact angle tests of the Col and Col-K foams in vertical direction and transverse direction.

The initiation and efficiency of coagulation closely corresponded to the blood absorption capacity of the materials. The parallel microstructure ensured that the material exhibited excellent directional liquid transport. We verified this using a methylene blue solution and blood (Fig. 8a and b). When the foam was placed perpendicularly onto the liquid surface, it showed the fastest liquid absorption rate in both the methylene blue solution and blood (Fig. 8c and d). The hydrophilicity of the parallel piping structures was further confirmed using a contact-angle instrument. In the transverse section, the water droplets immediately diffused into the foams. A slight delay occurred in the vertical section. The addition of kaolin enhanced the liquid absorption rate (Fig. 8e). Furthermore, the commercial gelatin sponge (HUSHIDA®) was also tested. However, after 20 min of soaking without external intervention, the material only exhibited surface wetness (Movie S5 and Fig. S3a). The dense film on the surface may be the main reason for blocking moisture.

Supplementary video related to this article can be found at https://doi.org/10.1016/j.bioactmat.2024.02.006

The following is the supplementary data related to this article:

Movie S5 Movie S5

When the porous pipe structure is in a dry state, liquid transmission mainly depends on the capillary force generated by the solid-liquid contact [48]. In general, the capillary pressure, interfacial tension, contact angle, and pore radius can be expressed as:

(1) $$\begin{array}{c} C_{p}=\frac{2\gamma \;\cos\;\theta }{a}\times A \end{array}$$

where *C*_p_ is the capillary pressure, *γ* is the interfacial tension, θ is the contact angle, *a* is the pore radius, and *A* is a constant (145 x 10^−3^).

Moreover, when the liquid flowed from the big pore toward the small pore in the gradient foams, the growing capillary pressure further accelerated the directional liquid transmission. Furthermore, similar to shorebirds, which obtain water by repeatedly opening and closing their V-shaped beaks [8,49], the Laplace pressure difference caused by the gradient change of the aperture is also the main force for directional liquid transport:

(2) $$\begin{array}{c} \Delta P=-\left(\frac{4\gamma \;\cos\;\theta }{X_{B}\alpha }-\frac{4\gamma \;\cos\;\theta }{X_{A}\alpha }\right) \end{array}$$

where *γ* is the liquid surface tension, *θ* is the contact angle between the liquid and solid, and *α* is the semi-apex angle of the cone. *X*_A_ and *X*_B_ are the distances of the liquid surface (sides A and B) to the apex of the cone.

In response to massive bleeding, foam can achieve rapid fluid absorption and activate blood coagulation at an early stage. In particular, in deep incompressible bleeding, the porous material is in close contact with blood. It can concentrate plasma and blood cells by absorbing excess water to promote hemostasis. Furthermore, the ability of the wound dressing to absorb fluid is an important factor in preventing excessive collection of wound exudates and avoiding secondary infections [50,51].

### 2.5 In vitro blood-clotting properties of the foams

**Fig. 9:**
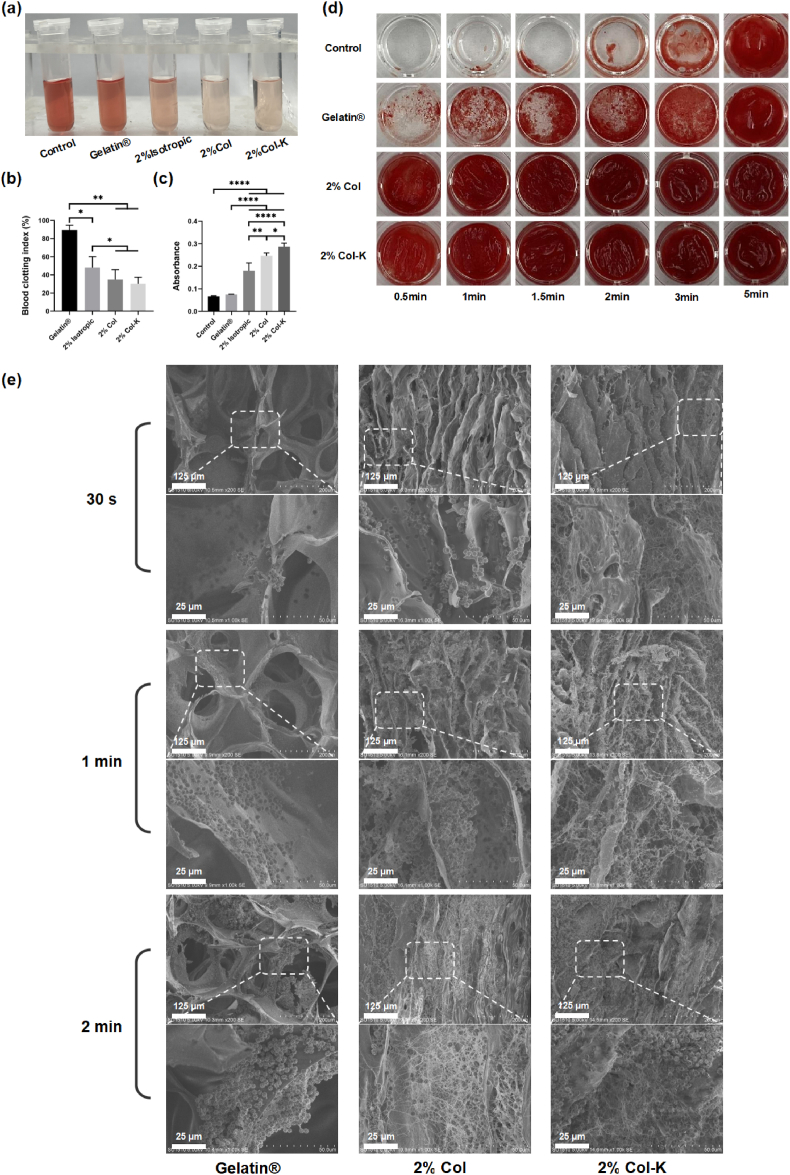
**In vitro hemostasis testing of the foams.** (a) Photographs from whole blood clotting assay of the foams using H_2_O as the negative control and Gelatin® as the positive control. (b) Whole blood clotting assay of different foams (n = 4). (c) Absorbance of LDH on different foams (n = 4). (d) Blood coagulation of foams at different time points. (e) SEM images of whole blood adhesion.

The whole blood clotting test is a widely accepted method for evaluating hemostatic properties in vitro, whereby a higher absorbance value of the hemoglobin solution indicates a slower clotting rate. Pure water was used as a negative control and Gelatin® as a positive control. In the context of blood-material interactions, it is evident that the supernatants of 2% Col and 2% Col-K exhibited a light red color, and 2% Isotropic a little darker. Gelatin® group displayed a darker red hue, which was more akin to the negative group (Fig. 9a). All collagen foams demonstrated lower blood clotting index (BCI) values compared to Gelatin® group, with 2% Col-K exhibiting the lowest BCI (27.47%) (Fig. 9b). For Gelatin®, the higher BCI probably resulted from its low absorption rate at the beginning (Fig. S3b). These findings showed that the aligned collagen foam had considerable blood-clotting properties, and the addition of kaolin further enhanced these advantages when the rate is concerned. Moreover, in order to further elucidate the structural contribution of collagen foams in coagulation, a comparative analysis was conducted between isotropic foams and anisotropic foams. These findings demonstrate that channeled collagen foams show obvious advantages in hemostasis, primarily attributed to their superior liquid permeability, larger contact area and more effective blood concentration (Fig. S4).

Platelet activation may enhance coagulation by exposing pro-coagulant surfaces, facilitating the generation of thrombin, and releasing proteins that aid the coagulation process. The lactate dehydrogenase (LDH) test was used to evaluate the platelet adhesion level on the foams by detecting the LDH produced from the lysed platelets [52]. Expectedly, these results suggested a sequential increase in platelet adhesion on Gelatin®, 2% Isotropic, 2% Col, and 2% Col-K (Fig. 9c). Furthermore, the adhesion and morphology of blood cells on the foams were examined to study their hemostatic mechanism, with Gelatin® sponges used as the control group. A recalcified whole-blood solution was added to the foam surface to simulate physiological bleeding (Fig. 9d). In the control group without any intervention, no obvious blood clots were observed until 5 min. Clotting was more pronounced in Gelatin® group, with a more complete hemagglutination layer formed at 3 min. In contrast, a large amount of blood cells was deposited in the 2% Col and 2% Col-K groups at 30 s, after which the clot component thickened over time. The 2% Col and 2% Col-K foams both showed excellent coagulation performance, and the differences were not distinguishable by the naked eye. To investigate the coagulation mechanism further, the surface of the material was observed using scanning electron microscopy. As seen in Fig. 9e, red blood cells (RBCs) started to assemble on Gelatin® group at 30s, but fibrin synthesis was not evident. After 1 and 2 min of contact with Gelatin®, some fibrin meshwork was sporadically visible. In contrast, an evident fibrin meshwork was present along with abundant RBCs aggregation at 30 s on collagen foams, owing to the high fluid absorption ability of the foam. The fibrin mesh density covering the RBCs increased after 1 and 2 min of interaction with the collagen foam. Compared to Gelatin®, 2% Col and 2% Col-K are not only liable to have platelets and red blood cells attached tightly but could also spontaneously trigger platelet activation and aggregation. Overall, the 2% Col-K group showed the highest fibrin mesh and blood cell attachment.

### 2.6 In vivo hemostatic properties of the foams

**Fig. 10:**
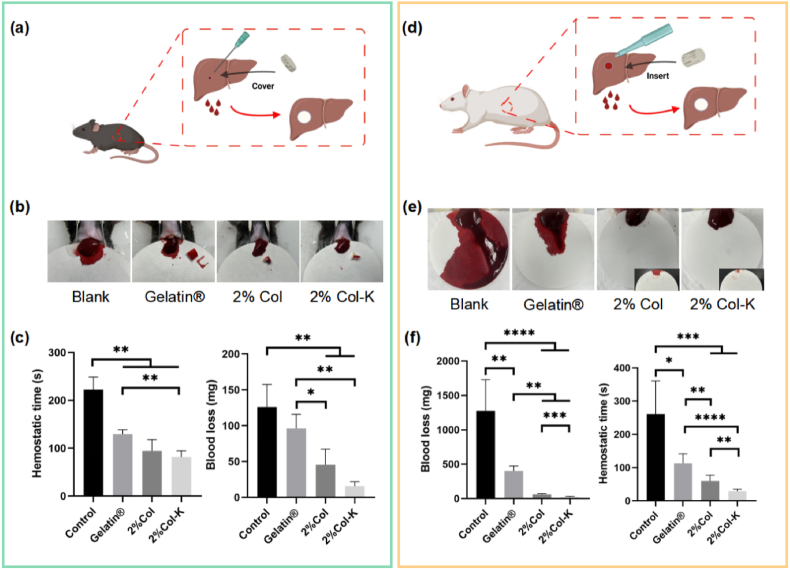
**In vivo hemostasis testing of the foams.** (a–c) Scheme illustration, blood loss and hemostatic time for the mouse liver bleeding model (n = 3). (d–f) Scheme illustration, blood loss and hemostatic time for the rat liver defect non-compressible hemorrhage model (n = 4–6).

Foams were evaluated for their hemostatic properties in a mouse liver injury model, measuring the amount of bleeding and hemostatic time to obtain foams with optimum hemostatic performance (Fig. 10a). Gelatin® sponge and the blank (no treatment) were used as the control. The results revealed that the trauma liver in the blank group lost 126.13 mg of blood within 222.67 s, while the Gelatin® groups showed blood losses of 96.27 mg and shorter bleeding times of 129.67 s. Compared with the controls, both aligned foam groups showed smaller blood losses of 45.5 and 15.7 mg, and a shorter hemostasis time of less than 100 s (94.33 and 81.67 s) with the addition of kaolin (Fig. 10b and c).

**Fig. 11:**
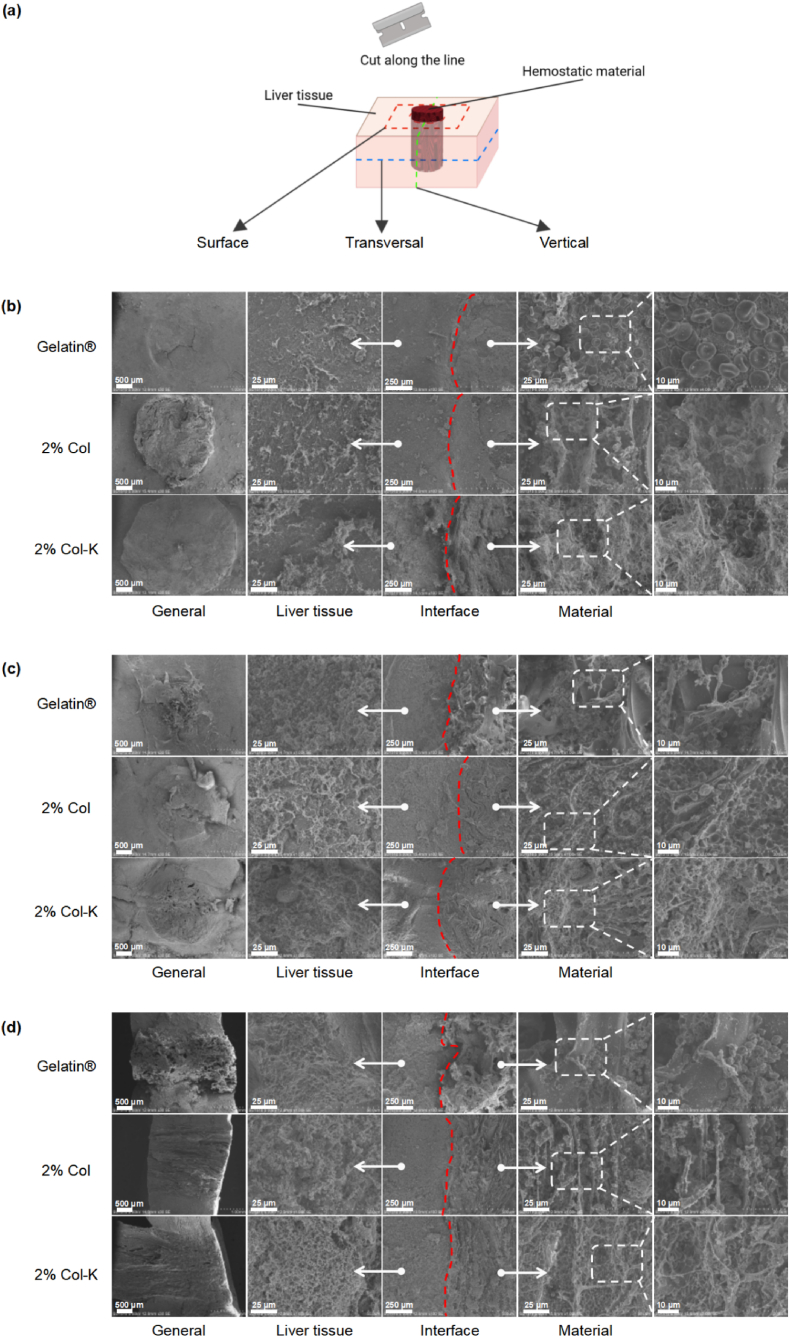
**Electron microscopy of the rat liver tissue.** (a) Schematic diagram of the observation area in composite material after hemostasis. (b) SEM observation of the surface in coagulation complexes. (c) SEM observation of transversal section in coagulation complexes. (d) SEM observation of vertical section in coagulation complexes.

However, hemorrhage in the mouse liver injury model was restricted and may not be enough. Therefore, the hemostatic potential of incompressible bleeding was further assessed using liver perforation. After inducing incompressible bleeding in the liver of a rat using a hole puncher, different materials were implanted into the site to determine their hemostatic performance (Fig. 10d and Movie S6–S9). The hemostatic times of the gelatin sponge, 2% Col foam, and 2% Col-K foam were 113.03, 59.85 and 29.19 s, respectively, while the blank control needed 260.86 s. Besides, the amount of bleeding after using the gelatin sponge, 2% Col foam, and 2% Col-K foam was 402.48, 60.65 and 22.08 mg, respectively, compared with the 1275.12 mg of the blank control (Fig. 10e and f). Subsequently, the coagulation complex was observed using SEM (Fig. 11a). On both the surface and transversal section (Fig. 11b and c), the most abundant fibrin network covering a number of blood cells, was found on 2% Col-K, indicating the formation of the most solid and mature blood clot. On the vertical section (Fig. 11d), it was observed that 2% Col and 2% Col-K closely fit the liver wound shape, while noticeable gap were present between the gelatin sponges and the liver tissue. These results revealed the outstanding hemostatic performance of the aligned foams, especially 2% Col-K. When inserted into the wound, the rapid liquid absorption of foams first led to blood-mediated shape recovery, resulting in compressive hemostasis. Especially in penetrating and gunshot injuries, such foams are expected to completely fill the irregular wound boundary in a short time and can greatly reduce bleeding in the form of inner compression. Meanwhile, the ordered pore structure ensures that the foam has appropriate mechanical properties and rapid liquid absorption, facilitating prompt interaction with the wound and blood. Concentrated blood, as well as the pro-coagulant substance in the foam, quickly activates the endogenous coagulation pathway as well as platelets [53,54]. A stable blood clot was eventually formed.

Supplementary video related to this article can be found at https://doi.org/10.1016/j.bioactmat.2024.02.006

The following are the supplementary data related to this article:

Movie S6 Movie S6

Movie S7 Movie S7

Movie S8 Movie S8

Movie S9 Movie S9

### 2.7 Biocompatibility of aligned foams

An ideal hemostatic material exhibits an excellent hemostatic performance and biocompatibility. The biocompatibility of 2% Col-K and 2% Col was investigated by assessing cytotoxicity, hemolysis, and histology.

#### 2.7.1 Hemolysis analysis

**Fig. 12:**
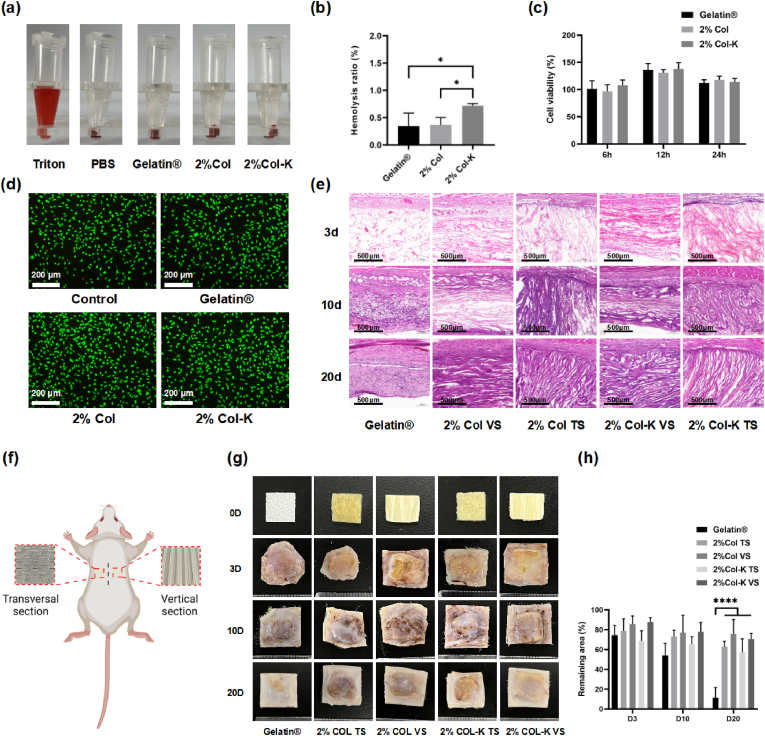
**Biocompatibility and biodegradation of aligned foams.** (a) Photographs from hemolytic activity assay of the foams using PBS as negative control and Triton X-100 as the positive control. (b) Hemolysis ratio of the foams at different concentrations (n = 5). (c) In vitro cytocompatibility of foams after incubation with the extract for 6, 12, and 24 h (n = 4). (d) LIVE/DEAD staining of L929 cells after incubation with the extract for 24 h (e) H&E staining of the embedded foams at day 3, 10, 20. (f) Scheme illustration for the rat subcutaneous implantation model. (g–h) Remaining area of the embedding foams on day 3, 10, and 20 after surgery (n = 4).

The in vitro hemolysis test is a standardized technical strategy for evaluating the hemocompatibility of materials. The hemolysis ratios of foams with various concentrations or additional kaolin were tested and compared with commercial gelatin sponges. Fig. 12 a illustrates the macroscopic color of supernatants after ultra-centrifugation for each foam group, the Gelatin® control group, the PBS negative group, and the Triton X-100 positive group. The positive group exhibited a vibrant red color, while the foams and Gelatin® groups displayed a pale-yellow hue akin to that of the PBS group. In Fig. 12b, despite the hemolysis ratio of 2% Col-K is marginally higher compared to Gelatin® and 2% Col, it remains significantly lower than 2%, as stipulated by ASTM F756-2017 [55] (nonhemolytic). This finding highlights the exceptional hemocompatibility of 2% Col-K as hemostatic agents or wound dressings.

#### 2.7.2 Cytotoxicity analysis

The Cell Counting Kit-8 (CCK-8) assay was used to examine the in vitro cytocompatibility of 2% Col and 2% Col-K, using Gelatin® as a positive control. Gelatin®, 2% Col, and 2% Col-K were non-toxic to L929 fibroblast cells after 6, 12, and 24 h of incubation, as shown in Fig. 12c. The biocompatibility of the foams was further confirmed through the 24-h live-dead staining results (Fig. 12d), as no discernible difference was observed between treated and control cells. Overall, 2% Col and 2% Col-K exhibited high cytocompatibility.

#### 2.7.3 Histology analysis

After subcutaneous implantation for 3, 10, and 20 days, the in vivo host response to the foams revealed a modest inflammatory reaction from H&E staining, which progressively diminished over time (Fig. 12e). Moreover, obvious inflammatory cell infiltration was observed at an early stage in the foam based on the orientation direction, which might help promote the rapid degradation and replacement of the scaffold with granulation tissue.

### 2.8 Biodegradation of the foams

The assessment of degradation performance is a crucial metric for biomaterials, necessitating varying timeframes depending on their intended application. Traditional collagen-based materials are limited by their susceptibility to degradation; however, our materials have made some improvement. Following a 20-day implantation period, the gelatin sponges had undergone near-complete degradation, whereas the 2% Col and 2% Col-K foams persisted to a certain extent (Fig. 12g and h). Given their biocompatibility and ability to facilitate cell ingrowth, these materials exhibited potential as highly effective, in vivo degradable hemostatic agents.

## 3 Conclusion

The ordered and gradient pore structure of the foams were designed using semiconductor modules. The orderly pattern ensured rapid liquid absorption during hemostatic application, allowing for prompt contact with the wound and blood and initiation of the subsequent endogenous coagulation pathway. The material's injectability and high recovery capacity confer upon it the ability to undergo blood-mediated shape recovery, thereby facilitating compression hemostasis.

## 4 Materials and methods

### 4.1 Materials

Collagen from pig skin (Mn = 300 kDa) was obtained from Wuxi Biot Biology Technology Co.Ltd., China. Kaolin was purchased from Shanghai Aladdin Biochemical Technology Co. Ltd., China. Acetic acid, glutaraldehyde, sodium citrate, calcium chloride, and anhydrous alcohol were purchased from Shanghai Sinopharm Chemical Reagent Co. Ltd., China. LDH release assay kit and Calcein/PI cell viability/cytotoxicity assay kit were purchased from Beyotime Biological Reagent Co. Ltd, Shanghai, China. The CCK8 Assay kit was purchased from MedChemExpress LLC, Shanghai, China. Gelatin sponges (HUSHIDA®, HSD-B) were obtained from Nanchang Hushida Medical Technology Co., Ltd, China. Sodium alginate, chitosan, gelatin, and polyvinyl alcohol were purchased from Sigma-Aldrich Co., LLC. All the components of the semiconductor freezing device were purchased from Alibaba and assembled in our laboratory. L929 cells (Cat. No. CCL-1) were obtained from American Type Culture Collection (ATCC).

### 4.2 Design and assembly of the freezing system

The freezing system mainly comprised a hydrocooling module, thermoelectric cooler, heat conduction board, and support generation module (Fig. 1, Fig. 2a). The thermoelectric cooler contained cooling and heating ends. A heat-conducting board was attached to the cooling end for rapid and uniform cooling, which was made from thermally conducive materials, such as silver, copper, or aluminum. A hydrocooling module was installed at the heating end to ensure heat dissipation and efficient cooling. The orientation support generation module comprised a heat insulation sleeve, a mold, and a sample pool. Finally, the thermostat achieved automatic temperature control by adjusting the output power through the real-time feedback of the temperature sensor connected to the cooling end.

### 4.3 Fabrication of the radially oriented foams

Common biological and synthetic materials have been used to test the wide applicability of the device. Sodium alginate or gelatin solutions were prepared by dissolving sodium alginate or gelatin in deionized water. Polyvinyl alcohol was dissolved in water at 90 °C, whereas chitosan was dissolved in acetic acid with a concentration of 0.1 M. After adjusting each solution to a final concentration of 2 wt%, they were added to a 10 mm high PVC sample pool that was wrapped in polystyrene foam as a thermal insulation material. The liquid surface was covered with heat-conducting plate with a temperature of **-**20°C for 4 h. The slurry was unidirectionally frozen from top to bottom under gravity. The infrared thermography (HIKMICRO, HM-TPH11-3AXF) was employed to observe the temperature distribution characteristics of materials. After 2 h of deeper cooling at **-**80°C, a freeze-dryer was used to lyophilize the crystallized materials for 48 h.

### 4.4 Fabrication of the collagen hemostatic foams

A series of different concentrations (0.5, 1, 2, and 4 wt%) were produced by dissolving collagen in 0.1 M acetic acid. After homogenization for 5 h at room temperature (20 °C), 0.5%, 1.5%, 2.5% (w/v) kaolin was added to the selected collagen solution (2 wt%). The solutions with and without kaolin were stored at 4 °C until use. Prior to lyophilization, the collagen solution was transformed into aligned hydrogels via mini-semiconductor modules. And isotropic collagen hydrogels were fabricated by uniformly freezing the collagen solution within a freezer set at −20 °C for 24 h. These foams were then cross-linked with glutaraldehyde vapor and sterilized with ^60^Co radiation before performing cell culture and in vivo experiments. Specifically, cross-linking was performed by saturating the foams in 25% glutaraldehyde in vapor phase at 20 °C for 24 h.

### 4.5 Characterization

The foams were cut in half in both the vertical and transverse sections and mounted on an aluminum sample stage. All the samples were sputter-coated with a thin layer of gold and observed using a scanning electron microscope (Hitachi SU1510) with an accelerating voltage of 5 kV. The pore distribution and mean channel width on the surface of the stubs were characterized using a nanometric software. Chemical structure analysis was performed using Fourier-transform infrared (FTIR) spectroscopy (Nicolet iS10, Thermo Fisher Scientific, USA).

The foam samples were weighed in a dry state and recorded as *M*_0_. The volume of foam was measured using a Vernier caliper and recorded as *V*. The mass–volume method was used to determine the apparent density (*ρ*) of the foams, and the following formula was used to calculate *ρ*:

(3) $$\begin{array}{c} \rho =\frac{M_{0}}{V} \end{array}$$

The porosity (*P*) values of the aligned foams were determined using a calibrated 10 mL pycnometer flask. The samples were immersed in a pycnometer flask with anhydrous alcohol for 30 min, followed by vacuum pumping to remove bubbles. The total weight of the bottle after filling it with alcohol was recorded as *M*_1_. In contrast, the pycnometer flask filled with ethanol was labeled *M*_2_. The apparent porosity (*P*) of the foams was determined using the following formula:

(4) $$\begin{array}{c} P=\frac{V\times \rho_{\text{alcohol}}-\left(M_{0}+M_{2}-M_{1}\right)}{V\times \rho_{\text{alcohol}}}\times 100\% \end{array}$$

The swelling ratio (*SR*) of the foams was determined using deionized water at room temperature (20 °C). Prior to the test, all the samples were dried in an oven to remove intrinsic water. The pre-weighed foams were placed in deionized water and air bubbles were removed using a vacuum pump. After 30 min of swelling equilibrium, the hydrated foams were removed and calculated as *M*_3_. The swelling ratios (*SR*) of the foams were determined using the following formulae:

(5) $$\begin{array}{c} SR=\frac{M_{3}-M_{0}}{M_{0}}\times 100\% \end{array}$$

### 4.6 Mechanical properties

To evaluate the mechanical properties of the foams, uniaxial and cyclic compression tests were performed with a universal electronic testing machine (WDW-1, Changzhou Sanfeng Instrument Technology Co., Ltd.). Before the compression test, foams cut into 10 mm cubes were incubated in deionized water to reach swelling equilibrium. The samples were then compressed at a rate of 3 mm/min and a maximum strain of up to 90% in both the vertical and horizontal directions. In the cyclic compression test, the same hydrous foam was squeezed at 3 mm/min to 80% strain and subsequently released at the same speed as that at 0% strain. This cycle was repeated ten times.

### 4.7 Shape memory and injectability

The aligned foam was incubated into deionized water and incubated at room temperature (20 °C) until equilibrium was reached. With the initial length recorded as *L*_1_, the hydrated foam was compressed to the maximum strain under the same stress in the direction perpendicular to the orientation, and then maintained for 1 min. After absorbing excess water, the length of the fixed foam was recorded as *L*_2_, the external force was removed, and the length of the stent 5 min later as *L*_3_. Finally, the foam was soaked in water for 1 min, and the recovery length was *L*_4_. Volume fixed ratio (*R*_f_), volume expansion ratio (*R*_e_) and shape recovery rate (*R*_r_) were calculated as follows:

(6) $$\begin{array}{c} R_{f}=\frac{L_{1}-L_{3}}{L_{1}}\times 100\% \end{array}$$

(7) $$\begin{array}{c} R_{e}=\frac{L_{4}}{L_{2}}\times 100\% \end{array}$$

(8) $$\begin{array}{c} R_{r}=\frac{L_{4}}{L_{1}}\times 100\% \end{array}$$

The initial, compressed, and recovery states of the foam were separately observed under a scanning electron microscope.

The aligned foam was prepared as a column with a diameter of 20 mm and a height of 10 mm. After full swelling in water, the foam was radically compressed to remove the free water within the foam matrix and loaded into a syringe (5 mm inner diameter). Then, the shape-fixed foam was injected into the water to restore its shape. Video and photographic recordings were performed during the injection.

### 4.8 Liquid absorption capacity and hydrophilicity

The aligned foams were cut into cubes with a height of 10 mm along the transverse and vertical directions, and isotropic collagen foam and gelatin foam were cut into the same size. Different materials were tested for their absorption rate of liquids with different viscosities (trypan blue solution and citrated blood). Moreover, a dynamic contact-angle instrument was employed to measure the dynamic droplet absorption progress for different orientations of the aligned foams. Finally, 2% Col and 2% Col-K were used to determine the effect of kaolin on absorption properties.

### 4.9 In vitro hemostatic assay

The samples were first cut into 10 mm cubes and preheated at 37 °C for 10 min. Following this, 100 μL of fresh anticoagulated whole mouse blood was placed onto the foam surface, accompanied by 20 μL of CaCl_2_ (0.2 M) solution. The negative control (reference value) was established by directly introducing 100 μL of blood and 20 μL of CaCl_2_ solution into 10 mL pure water. Following a 10-min incubation period at 37 °C, 10 mL of pure water was gradually introduced into the beaker, without disrupting the clotted blood, and subsequently, the mixture was subjected to incubation on a shaker table at 37 °C and 30 rpm for another 10 min. The uncoagulated RBCs swelled and burst in water, releasing hemoglobin [56]. The Gelatin® foam was subjected to the same treatment as the positive control. Using a microplate reader, the absorbance of each sample in the hemoglobin solution was measured at 540 nm. The BCI is presented as follows:

(9) $$\begin{array}{c} \text{BCI}=\frac{I_{s}}{I_{w}}\times 100\% \end{array}$$

where *I*_s_ is the absorbance of the sample and *I*_w_ is the absorbance of the reference value.

### 4.10 Clotting time assay

The clotting time assay was performed in accordance with a previously reported protocol [57]. Firstly, 0.1 M CaCl_2_ was added to citrated blood in a 1:9 ratio and mixed for 10 s. The mixture was then added to a 96-well plate to a volume of 50 μL. Each well was washed with saline solution (9 g/L) at predetermined time intervals (0.5min, 1min, 1.5min, 2min, 3min and 5min) to prevent clotting. The procedure was repeated three times to remove physically adherent blood components. When testing the aligned foams, all were cut into slices of the same thickness to exactly cover the bottom layer of the plate. Gelatin® foam was used as a positive control. After fixation with 2.5% glutaraldehyde and dehydration in a graded series of *tert*-butyl alcohol solutions (50, 75, 80, 90, and 100%), the lyophilized coagulation complexes on the foam were also observed by SEM.

### 4.11 Platelet adhesion

The LDH test was first performed to measure the LDH produced from lysed platelets to evaluate the adhesion level of platelets on collagen foam. Platelet-rich plasma was acquired through the centrifugation of citrated whole blood at 2000 rpm for 10 min. Then, 100 μL of platelet-rich plasma was added to the foam with 0.5 cubic centimeter and incubated at 37 °C for 30 min. Afterwards, the sample was dipped and rinsed in PBS to wash away the non-adherent platelets, and then incubated with 1% Triton X-100 at 37 °C for 1 h to lyse the adherent platelets. Gelatin® foam was used as a positive control, and adherent platelets were analyzed using an LDH kit according to the manufacturer's instructions.

### 4.12 In vivo hemostatic properties

Both the mouse liver bleeding model and rat liver volume defect model were used to assess the hemostatic capacity of the foams. C57BL/6 mice (7–8 weeks-old males, weighing 20 g) were derived from Cavens Biogle (Suzhou, China) Model Animal Research Co.Ltd. Sprague−Dawley (S-D) rats (6–8 weeks-old males, weighing 200–220 g) were procured from Changzhou Cavens Lab Animal Co., Ltd. (Changzhou, China). All animal experiments were conducted in compliance with the regulations sanctioned by the Jiangnan University Animal Research Committee. (Protocol JN.No 20210415c0300631 and JN.No 20220930S0380115).

The mice used for the liver bleeding experiments (n = 20 mice) were randomly and equally allocated into four groups. The liver of each mouse was exposed through an abdominal incision, and to ensure accurate estimation of the weight of blood obtained from hemostatic samples, the serous fluid surrounding the liver was meticulously removed. A pre-weighed filter paper was placed between a plastic film and the liver, and the liver of the mouse was punctured to bleed with a 16G needle on a 30-degree inclined plate. Gelatin® and collagen foams were promptly applied to the bleeding site, and hemostatic time and blood loss were recorded during hemostasis. Five mice were included in each group.

The rat liver volume defect was used as a model of non-compressible hemorrhagic hemostasis to assess the hemostatic capacity of the aligned foams. For further in vivo injection, the sterilized foam (5 mm in diameter and 5 mm in height) was compressed to a diameter of 2 mm. S-D rats were divided equally and randomly into 4 groups. Following fixation on the surgical corkboard, the animals were anesthetized with 3% sodium amobarbital (2 mL/100 g). Subsequently, the rats were given an abdominal incision to reveal the liver. After removing excess serous fluid, a columniform defect in the liver volume (2 mm in diameter and 5 mm in height) was formed in the right lobe using surgical scissors and a biopsy needle with an inner diameter of 2 mm. The foam was immediately inserted into the defected orifice. The non-intervention and gelatin® foams served as the control groups. During the hemostatic procedure, the pre-weighed gauze was utilized to absorb the flowing blood. The blood loss, hemostatic time, and vital state were noted.

Four replicates were performed for each group. Histological analyses were performed as previously described. Briefly, paraformaldehyde-fixed (6 μm) cryosections from all explants were stained with hematoxylin/eosin and analyzed microscopically.

### 4.13 Hemolysis assay

For the hemolysis assay, erythrocytes were obtained from fresh anticoagulated whole blood of mice by centrifuging at 2000 rpm and 4 °C for 10 min. The erythrocytes obtained were washed several times with PBS until the supernatant became colorless and transparent. After purification, erythrocytes were diluted with PBS to achieve a final concentration of 5% (v/v). The erythrocyte suspension (300 μL) and foam powder (5 mg; prepared using a grinder) were added to 1 mL PBS. Positive and negative controls were utilized, employing Triton X-100 and PBS, respectively. The samples were maintained at 37 °C for 2 h and subsequently centrifuged at 2000 rpm for 5 min. The absorbance of the supernatant was measured at 540 nm using a microplate reader. The hemolysis ratio of the foams was calculated as follows:

(10) $$\begin{array}{c} \text{Hemolysis}\;\text{ratio}=\frac{A_{d}-A_{f}}{A_{e}-A_{f}}\times 100\% \end{array}$$

where *A*_d_ is the absorbance of the supernatant in the sample groups, *A*_e_ is the absorbance of the Triton X-100 positive control group, and *A*_f_ is the absorbance of PBS.

### 4.14 In vitro cytotoxicity

A CCK-8 assay was performed to evaluate the cytotoxicity of the foams in L929 cells (a mouse fibroblast cell line). All the foams were placed in PBS at a density of 10 mg/mL prior to the test, and the extract was then produced after a 24-h incubation at 37 °C. Before switching the culture media to 100 μL fresh medium supplemented with 20 μL extract solutions, L929 cells were seeded in 96-well plates at a density of 3000 cells/well in 100 μL of DMEM medium (with 10% fetal bovine serum and 1% penicillin-streptomycin) and pre-cultured at 37 °C under 5% humidified CO_2_ for 12 h. After 6 h, 12 h and 24 h, the cytotoxicity of the foams was assessed using Cell Counting KIT-8 (CCK-8) at an optical density (OD) of 450 nm and then evaluated by Calcein/PI cell viability/cytotoxicity assay kit at 24 h. Cell viability was calculated as follows:

(11) $$\begin{array}{c} \text{Cell}\;\text{viability}=\frac{A_{a}-A_{c}}{A_{b}-A_{c}}\times 100\% \end{array}$$

where *A*_a_ is the optical density of the well with L929 cells and extract solutions, *A*_b_ is the optical density of the well with L929 cells, and *A*_c_ is the optical density of the well without L929 cells.

### 4.15 In vivo biodegradation

A subcutaneous rat implantation model was used to assess the biocompatibility of the foams. Prior to implantation, all materials were first cut to the same size (10 mm diameter and 2 mm height), sterilized with 75% ethanol, and finally washed several times with PBS. Dorsal skin incisions (1 cm in length) were made on the same area on the back of each rat after anesthesia. After placement, the test articles were implanted subcutaneously into the incision and the skin was closed anatomically. Animals were euthanized after 3, 10, and 20 d by CO_2_ inhalation, and implants along with adjacent tissues were digitally recorded and processed for histological analysis.

### 4.16 Statistical analysis

All quantitative data are reported as the mean ± standard deviation. To analyze statistic differences among groups, one-way ANOVA and Student's T test were conducted, with a significance level of P = 0.05. Significance levels are denoted as follows: P < 0.05 (*), P < 0.01 (**), P < 0.001 (***), and P < 0.0001 (****).

## Ethics

All animal experiments were conducted in compliance with the regulations sanctioned by the Jiangnan University Animal Research Committee. (Protocol JN.No 20210415c0300631 and JN.No 20220930S0380115).

## Declaration of interest

All authors declare there is no conflict of interest in this manuscript.

## CRediT authorship contribution statement

**Fengbo Yang:** Writing – original draft, Validation, Methodology, Investigation, Conceptualization. **Xiaoli Jia:** Writing – review & editing, Validation, Methodology, Investigation. **Chao Hua:** Writing – review & editing, Visualization, Validation, Methodology, Investigation. **Feifan Zhou:** Visualization, Validation, Methodology. **Jianing Hua:** Visualization, Validation, Investigation. **Yuting Ji:** Visualization, Validation, Methodology. **Peng Zhao:** Validation, Project administration, Methodology. **Quan Yuan:** Methodology, Validation, Writing – review & editing. **Malcolm Xing:** Writing – review & editing, Writing – original draft, Supervision, Project administration, Methodology, Conceptualization. **Guozhong Lyu:** Writing – review & editing, Supervision, Project administration, Methodology, Investigation.

## References

[1] Feng C., Zhang W., Deng C., Li G., Chang J., Zhang Z. (2017). 3D printing of lotus root-like biomimetic materials for cell delivery and tissue regeneration. Adv. Sci..

[2] Liu D., Lei C., Wu K., Fu Q. (2020). A multidirectionally thermoconductive phase change material enables high and durable electricity real-environment solar-thermal-electric conversion. ACS Nano.

[3] Matthews P.G., Seymour R.S. (2014). Stomata actively regulate internal aeration of the sacred lotus Nelumbo nucifera. Plant Cell Environ..

[4] Fan S., Wu X., Fang Z., Yang G., Yang J., Zhong W., Luo J., Xing M., Wan W. (2023). Injectable and ultra-compressible shape-memory mushroom: highly aligned microtubules for ultra-fast blood absorption and hemostasis. Chem. Eng. J..

[5] Dudukovic N.A., Fong E.J., Gemeda H.B., DeOtte J.R., Cerón M.R., Moran B.D. (2021). Cellular fluidics. Nature.

[6] Xu K., Wu X., Zhang X., Xing M. (2022). Bridging wounds: tissue adhesives’ essential mechanisms, synthesis and characterization, bioinspired adhesives and future perspectives. Burns & Trauma.

[7] Afshar M., Pourkamali Anaraki A., Montazerian H. (2018). Compressive characteristics of radially graded porosity scaffolds architectured with minimal surfaces. Mater. Sci. Eng. C.

[8] Dai H., Dong Z., Jiang L. (2020). Directional liquid dynamics of interfaces with superwettability. Sci. Adv..

[9] Joukhdar H., Seifert A., Jüngst T., Groll J., Lord M.S., Rnjak-Kovacina J. (2021). Ice templating soft matter: fundamental principles and fabrication approaches to tailor pore structure and morphology and their biomedical applications. Adv. Mater..

[10] McLinden M.O., Seeton C.J., Pearson A. (2020). New refrigerants and system configurations for vapor-compression refrigeration. Science.

[11] Evich M.G., Davis M.J.B., McCord J.P., Acrey B., Awkerman J.A., Knappe D.R.U. (2022). Per- and polyfluoroalkyl substances in the environment. Science.

[12] Council N.R. (2011).

[13] Xia Y., Mathis T.S., Zhao M.Q., Anasori B., Dang A., Zhou Z. (2018). Thickness-independent capacitance of vertically aligned liquid-crystalline MXenes. Nature.

[14] Shen X., Zheng Q., Kim J.-K. (2021). Rational design of two-dimensional nanofillers for polymer nanocomposites toward multifunctional applications. Prog. Mater. Sci..

[15] Li Q., Yuan Z., Zhang C., Hu S., Chen Z., Wu Y. (2022). Tough, highly oriented, super thermal insulating regenerated all-cellulose sponge-aerogel fibers integrating a graded aligned nanostructure. Nano Lett..

[16] Ma J., Lin S., Jiang Y., Li P., Zhang H., Xu Z. (2020). Digital programming graphene oxide liquid crystalline hybrid hydrogel by shearing microlithography. ACS Nano.

[17] Cao M., Li Z., Lu J., Wang B., Lai H., Li Z. (2023). Vertical array of graphite oxide liquid crystal by microwire shearing for highly thermally conductive composites. Adv. Mater..

[18] Ge Y.W., Chu M., Zhu Z.Y., Ke Q.F., Guo Y.P., Zhang C.Q. (2022). Nacre-inspired magnetically oriented micro-cellulose fibres/nano-hydroxyapatite/chitosan layered scaffold enhances pro-osteogenesis and angiogenesis. Materials today Bio.

[19] Ding B., Zeng P., Huang Z., Dai L., Lan T., Xu H. (2022). A 2D material-based transparent hydrogel with engineerable interference colours. Nat. Commun..

[20] Zhu Q.L., Du C., Dai Y., Daab M., Matejdes M., Breu J. (2020). Light-steered locomotion of muscle-like hydrogel by self-coordinated shape change and friction modulation. Nat. Commun..

[21] Chen Y., Liu Y., Xia Y., Liu X., Qiang Z., Yang J. (2020). Electric field-induced assembly and alignment of silver-coated cellulose for polymer composite films with enhanced dielectric permittivity and anisotropic light transmission. ACS Appl. Mater. Interfaces.

[22] Wu W., Liu X., Qiang Z., Yang J., Liu Y., Huai K. (2021). Inserting insulating barriers into conductive particle channels: a new paradigm for fabricating polymer composites with high dielectric permittivity and low dielectric loss. Compos. Sci. Technol..

[23] Gokcekaya O., Hayashi N., Ishimoto T., Ueda K., Narushima T., Nakano T. (2020). Crystallographic orientation control of pure chromium via laser powder bed fusion and improved high temperature oxidation resistance. Addit. Manuf..

[24] Tan L.J., Zhu W., Zhou K. (2020). Recent progress on polymer materials for additive manufacturing. Adv. Funct. Mater..

[25] Ribeiro I., Matos F., Jacinto C., Salman H., Cardeal G., Carvalho H. (2020). Framework for life cycle sustainability assessment of additive manufacturing. Sustainability.

[26] Zhang D., Liu W., Guo R., Zhou K., Luo H. (2018). High discharge energy density at low electric field using an aligned titanium dioxide/lead zirconate titanate nanowire array. Adv. Sci..

[27] Yao B., Chen J., Huang L., Zhou Q., Shi G. (2016). Base-induced liquid crystals of graphene oxide for preparing elastic graphene foams with long-range ordered microstructures. Adv. Mater..

[28] Lin Y., Chen J., Jiang P., Huang X. (2020). Wood annual ring structured elastomer composites with high thermal conduction enhancement efficiency. Chem. Eng. J..

[29] An F., Li X., Min P., Li H., Dai Z., Yu Z.-Z. (2018). Highly anisotropic graphene/boron nitride hybrid aerogels with long-range ordered architecture and moderate density for highly thermally conductive composites. Carbon.

[30] Ping L., Hou P.-X., Liu C., Cheng H.-M. (2019). Vertically aligned carbon nanotube arrays as a thermal interface material. Apl. Mater..

[31] Hua M., Wu S., Ma Y., Zhao Y., Chen Z., Frenkel I. (2021). Strong tough hydrogels via the synergy of freeze-casting and salting out. Nature.

[32] Chan K.-Y., Shen X., Yang J., Lin K.-T., Venkatesan H., Kim E. (2022). Scalable anisotropic cooling aerogels by additive freeze-casting. Nat. Commun..

[33] Cheng Y., Li X., Qin Y., Fang Y., Liu G., Wang Z. (2021). Hierarchically porous polyimide/Ti_3_C_2_T_x_ film with stable electromagnetic interference shielding after resisting harsh conditions. Sci. Adv..

[34] Peltier J.C.A. (1834). Nouvelles expériences sur la caloricité des courants électrique. Ann. Chim. Phys..

[35] Kishore R., Nozariasbmarz A., Poudel B., Sanghadasa M., Priya S.J. (2019). Ultra-high performance wearable thermoelectric coolers with less materials. Nat. Commun..

[36] Mao J., Chen G., Ren Z. (2021). Thermoelectric cooling materials. Nat. Mater..

[37] King D.R. (2019). Initial care of the severely injured patient. N. Engl. J. Med..

[38] Alarhayem A.Q., Myers J.G., Dent D., Liao L., Muir M., Mueller D. (2016). Time is the enemy: mortality in trauma patients with hemorrhage from torso injury occurs long before the “golden hour. Am. J. Surg..

[39] Wang L., Zhong Y., Qian C., Yang D., Nie J., Ma G. (2020). A natural polymer-based porous sponge with capillary-mimicking microchannels for rapid hemostasis. Acta Biomater..

[40] Michel R., Poirier L., van Poelvoorde Q., Legagneux J., Manassero M., Corté L. (2019). Interfacial fluid transport is a key to hydrogel bioadhesion. Proc. Natl. Acad. Sci. U. S. A..

[41] Chen P., Tao J., Zhu S., Cai Y., Mao Q., Yu D. (2015). Radially oriented collagen scaffold with SDF-1 promotes osteochondral repair by facilitating cell homing. Biomaterials.

[42] Shao G., Hanaor D.A.H., Shen X., Gurlo A. (2020). Freeze casting: from low-dimensional building blocks to aligned porous structures—a review of novel materials, methods, and applications. Adv. Mater..

[43] Deville S., Saiz E., Nalla R.K., Tomsia A.P. (2006). Freezing as a path to build complex composites. Science.

[44] Yan T., Cheng F., Wei X., Huang Y., He J. (2017). Biodegradable collagen sponge reinforced with chitosan/calcium pyrophosphate nanoflowers for rapid hemostasis. Carbohydr. Polym..

[45] Tamer T.M., Sabet M.M., Omer A.M., Abbas E., Eid A.I., Mohy-Eldin M.S. (2021). Hemostatic and antibacterial PVA/Kaolin composite sponges loaded with penicillin-streptomycin for wound dressing applications. Sci. Rep..

[46] Raji Vijay V., Anitha A.M., Ravindranatha Menon A.R. (2016). Studies on blends of natural rubber and butadiene rubber containing silica—organomodified kaolin hybrid filler systems. Polymer.

[47] Ge X., Chang M., Jiang W., Zhang B., Xing R., Bulin C. (2020). Selective location of kaolin and effects of maleic anhydride in kaolin/poly(ε-caprolactone)/poly(lactic acid) composites. Appl. Clay Sci..

[48] Chen H., Ran T., Gan Y., Zhou J., Zhang Y., Zhang L. (2018). Ultrafast water harvesting and transport in hierarchical microchannels. Nat. Mater..

[49] Cui Y., Li D., Bai H. (2017). Bioinspired smart materials for directional liquid transport. Ind. Eng. Chem. Res..

[50] Landsman T.L., Touchet T., Hasan S.M., Smith C., Russell B., Rivera J. (2017). A shape memory foam composite with enhanced fluid uptake and bactericidal properties as a hemostatic agent. Acta Biomater..

[51] Zhao X., Guo B., Wu H., Liang Y., Ma P.X. (2018). Injectable antibacterial conductive nanocomposite cryogels with rapid shape recovery for noncompressible hemorrhage and wound healing. Nat. Commun..

[52] Yang X., Liu W., Shi Y., Xi G., Wang M., Liang B. (2019). Peptide-immobilized starch/PEG sponge with rapid shape recovery and dual-function for both uncontrolled and noncompressible hemorrhage. Acta Biomater..

[53] Deng Y., Yang X., Zhang X., Cao H., Mao L., Yuan M. (2020). Novel fenugreek gum-cellulose composite hydrogel with wound healing synergism: facile preparation, characterization and wound healing activity evaluation. Int. J. Biol. Macromol..

[54] Long M., Zhang Y., Huang P., Chang S., Hu Y., Yang Q. (2018). Emerging nanoclay composite for effective hemostasis. Adv. Funct. Mater..

[55] (2017). ASTM F756-17: standard practice for assessment of hemolytic properties of materials.

[56] Jia X., Hua C., Yang F., Li X., Zhao P., Zhou F. (2023). Hydrophobic aerogel-modified hemostatic gauze with thermal management performance. Bioact. Mater..

[57] Guo Y., Wang Y., Zhao X., Li X., Wang Q., Zhong W. (2021). Snake extract-laden hemostatic bioadhesive gel cross-linked by visible light. Sci. Adv..

