# Supplementary material for: Highly efficient semiconductor modules making controllable parallel microchannels for non-compressible hemorrhages
Source: Bioact Mater. 2024 Feb 23;36:30–47. doi: 10.1016/j.bioactmat.2024.02.006 (PMC10904172; doi:10.1016/j.bioactmat.2024.02.006)
Supplement: Multimedia component 1 [file mmc1.docx]

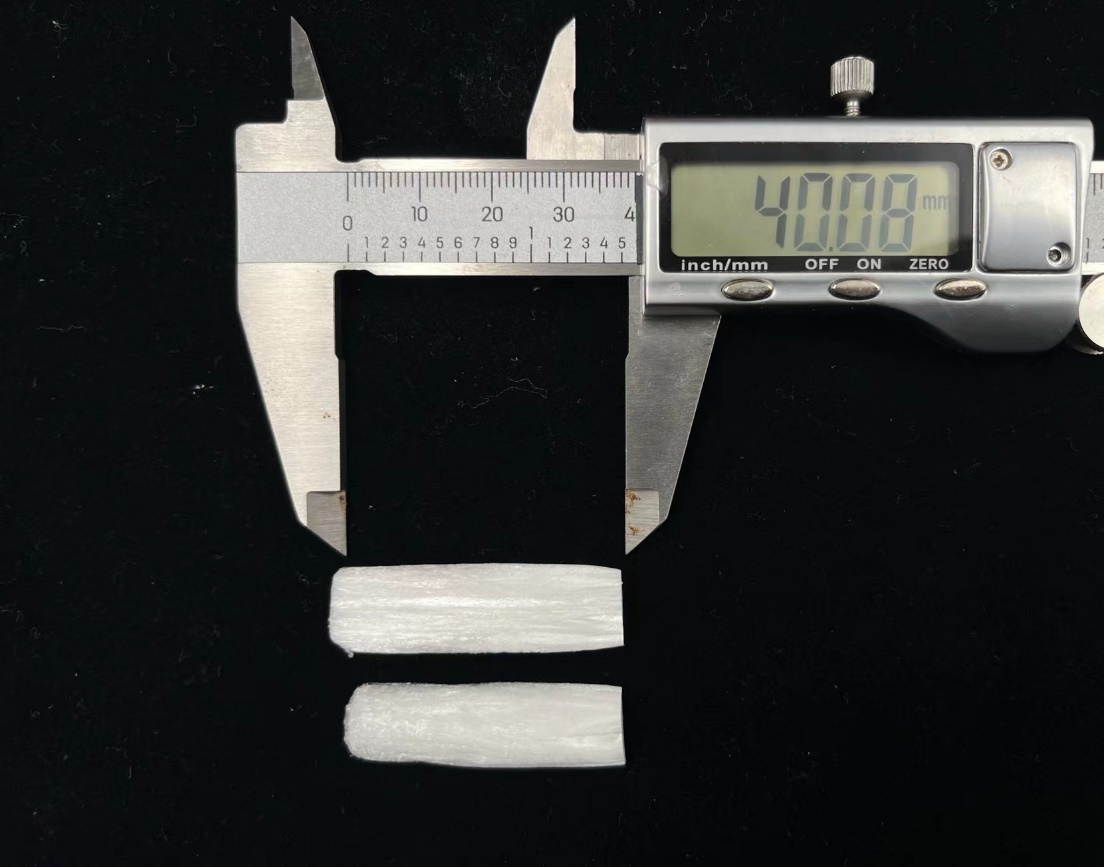


**Fig. S1. Long paralleled microtubular foams.**


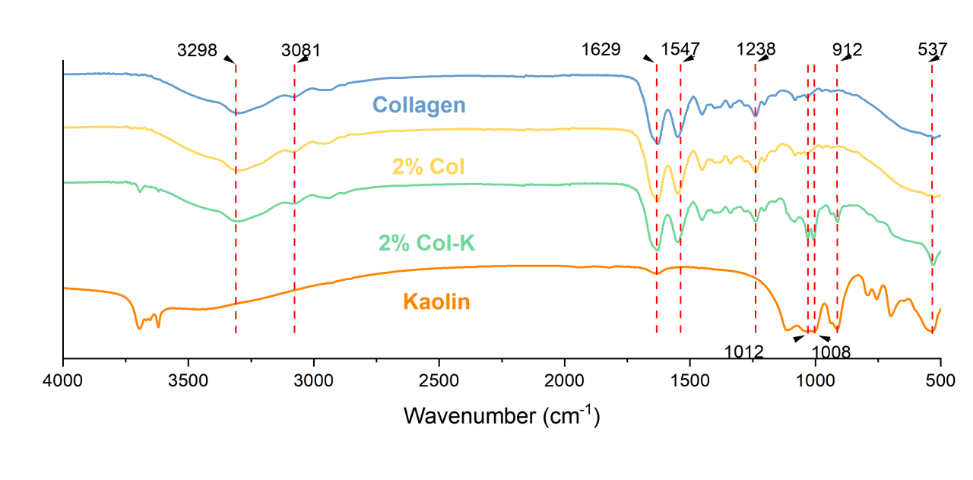


**Fig. S2. FTIR of kaolin powder, soluble collagen foam, 2% Col foam and 2% Col-K foam.**

**
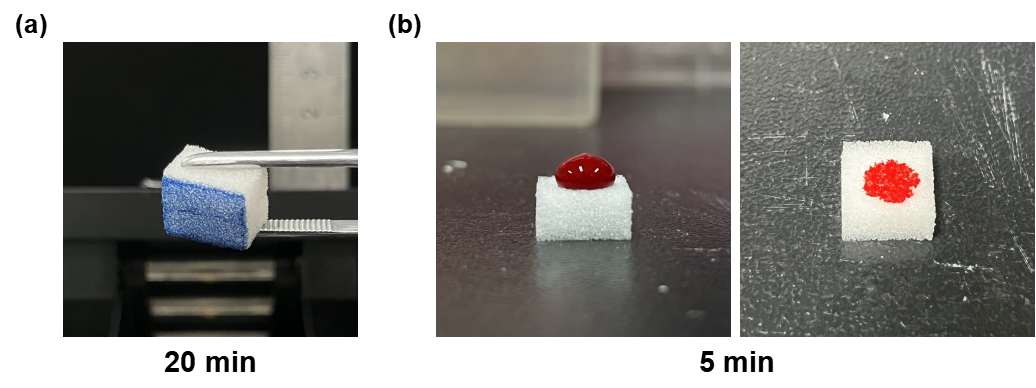
**

**Fig. S3. Wettability characteristics of the Gelatin® foam.** (a) Gelatin® foam immersed in methylene blue solution for 20 minutes. (b) Gelatin® foam contacted with blood for 5 minutes and removed of excess blood.


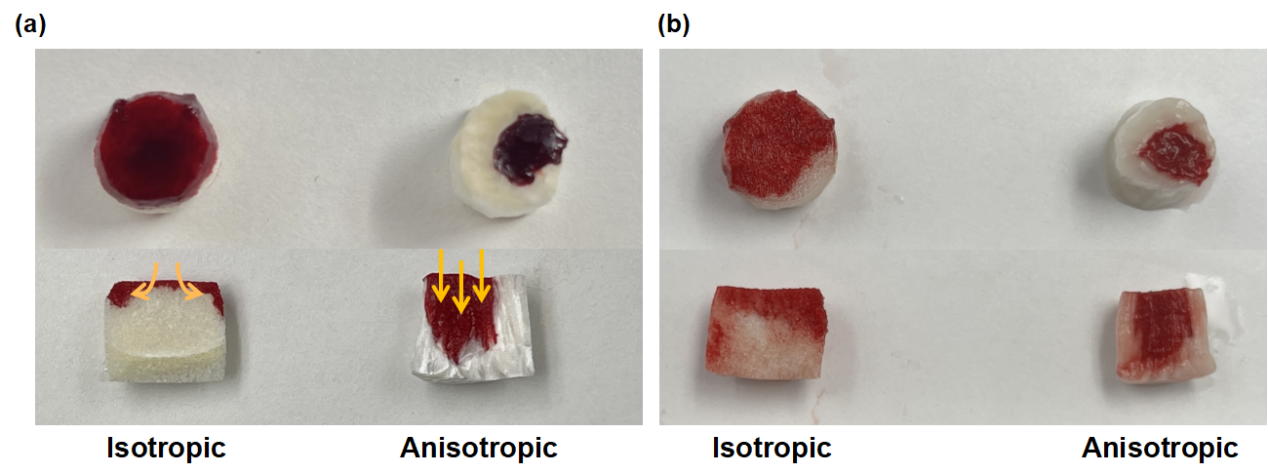


**Fig. S4. In vitro blood coagulation with isotropic collagen foam (2% Isotropic) and anisotropic collagen foam (2% Col).** (a) After five minutes of contact with blood, isotropic collagen foam and anisotropic collagen foam were observed. (b) The isotropic collagen foam and anisotropic collagen foam were observed after the non-adherent blood cells were removed by rinsing.

**List of supplementary movie information**

Movie S1. Rapid freezing process of collagen droplets on common thermoelectric semiconductor module (5X).

Movie S2. Rapid freezing process of collagen droplets on circular thermoelectric semiconductor module (5X).

Movie S3. Shape memory test of the aligned foams (10X).

Movie S4. Injection performance test of the aligned foams.

Movie S5. Movie of wettability characteristics of the Gelatin® foam (100X).

Movie S6. Hemostatic test of non-treatment on rat liver defect non-compressible hemorrhage model (20X).

Movie S7. Hemostatic test of Gelatin® foam on rat liver defect non-compressible hemorrhage model (20X).

Movie S8. Hemostatic test of 2% Col foam on rat liver defect non-compressible hemorrhage model (20X).

Movie S9. Hemostatic test of 2% Col-K foam on rat liver defect non-compressible hemorrhage model (20X).
